# ABPP-HT - High-Throughput Activity-Based Profiling of Deubiquitylating Enzyme Inhibitors in a Cellular Context

**DOI:** 10.3389/fchem.2021.640105

**Published:** 2021-02-25

**Authors:** Hannah B. L. Jones, Raphael Heilig, Roman Fischer, Benedikt M. Kessler, Adán Pinto-Fernández

**Affiliations:** Nuffield Department of Medicine, Target Discovery Institute, Centre for Medicines Discovery, University of Oxford, Oxford, United Kingdom

**Keywords:** activitomics, activity-based probes, deubiquitylating enzymes, ubiquitin, proteomics, drug discovery, pharmacology, chemical biology

## Abstract

The potency and selectivity of a small molecule inhibitor are key parameters to assess during the early stages of drug discovery. In particular, it is very informative for characterizing compounds in a relevant cellular context in order to reveal potential off-target effects and drug efficacy. Activity-based probes are valuable tools for that purpose, however, obtaining cellular target engagement data in a high-throughput format has been particularly challenging. Here, we describe a new methodology named ABPP-HT (high-throughput-compatible activity-based protein profiling), implementing a semi-automated proteomic sample preparation workflow that increases the throughput capabilities of the classical ABPP workflow approximately ten times while preserving its enzyme profiling characteristics. Using a panel of deubiquitylating enzyme (DUB) inhibitors, we demonstrate the feasibility of ABPP-HT to provide compound selectivity profiles of endogenous DUBs in a cellular context at a fraction of time as compared to previous methodologies.

## Introduction

Activity-based probes (ABPs) can assess enzyme activity and inhibition within a cellular environment, thereby providing a considerable advantage over classical biochemical and enzyme assays. ABPs typically consist of a reactive group that binds to the active site of an enzyme, mostly in a covalent fashion, a specific binding group/linker to aid target binding/prevent steric hindrance, and a reporter tag for fluorescence or affinity (Chen et al., 2017; Chakrabarty et al., 2019; Deng et al., 2020). ABPs with an electrophilic reactive group can be applied to multiple enzymes including serine hydrolases, kinases, metalloproteases and cysteine proteases (Niphakis and Cravatt, 2014). Where a nucleophilic active site does not exist, photo-affinity probes can be applied instead (Niphakis and Cravatt, 2014; Mathur et al., 2020). A very informative application of these probes is the activity-based protein profiling (ABPP), combining labeling with ABPs, immunoaffinity purification, and mass spectrometry (IAP-MS) to analyze the probe interactome (Benns et al., 2021). ABPP has been used to study the potency and selectivity of small molecule inhibitors in an unbiased manner, in a cellular matrix (Nguyen et al., 2017; Wang et al., 2018).

Profiling the ubiquitin conjugating activity of E3 ubiquitin ligases and deubiquitylating enzymes (DUBs) is of significant interest due to post-translational protein ubiquitination regulating numerous cellular pathways. These include protein degradation, localization or controlling function (Hershko and Ciechanover, 1992; Mukhopadhyay and Riezman, 2007). Dysregulation of ubiquitination has been linked to several pathologies including cancers and neurodegenerative diseases (Popovic et al., 2014). Consequently, various E3 ligases and DUBs are being evaluated as potential drug targets for either protein targeting chimeras (PROTACs) or inhibitors, respectively, due to their proven ability to specifically target cellular protein homeostasis (Huang and Dixit, 2016; Lai and Crews, 2017; Harrigan et al., 2018).

DUBs offer a mechanistic entry point for probe targeting as the majority are cysteine proteases, with a smaller subsection (<15%) functioning as metalloproteases (Komander et al., 2009; Clague et al., 2019). The activity of thiol protease DUBs can be ascertained via covalent attachment of an electrophile to their nucleophilic active site within the catalytic domain. This was first achieved by replacing the C-terminal glycine 76 of ubiquitin with glycyl vinyl sulfone (Borodovsky et al., 2001), and further developed toward a panel of seven different Ub probes with different electrophilic warheads (Borodovsky et al., 2002). These studies have provided the framework for expanding the ubiquitin-based probe concept regarding synthesis (El Oualid et al., 2010) and chemical capture (Hewings et al., 2017), but also targeting metallo-DUBs (Hameed et al., 2019) and E3 ligases (Mulder et al., 2016; Pao et al., 2016).

The inclusion of an affinity tag such as hemagglutinin (HA), FLAG, biotin, etc. to the N-terminus of a ubiquitin-based probe allows for DUB enrichment by immunoprecipitation (IP). Subsequent analysis by liquid chromatography tandem mass spectrometry (LC-MS/MS) can identify and quantify cellular active DUBs bound to the probe. This method can be used in conjunction with a DUB inhibitor to identify inhibitor potency and cross-reactivity. Any DUBs that react with the inhibitor will not bind to the probe as efficiently and will be reduced in the immunoprecipitated sample when compared to a control. This method was successfully applied to demonstrate the selectivity of a USP7 inhibitor, with a 2-bromoethylamine warhead probe (HA-Ub-Br2). In this case, a panel of 22 DUBs were quantified (Turnbull et al., 2017). The ABPP assay was used also to assess cellular target engagement and DUB selectivity in crude cell extracts for small molecule inhibitors against USP9X (Clancy et al., 2020), and USP28 (Ruiz et al., 2020).

Without fractionation the number of DUBs immunoprecipitated and quantified by MS with a propargylamine (PA) warhead is around 30–40 (Altun et al., 2011; Ekkebus et al., 2013). To improve this methodology, we have recently combined this probe (Ub-PA) with sample fractionation and 74 DUBs in MCF-7 breast cancer cells were quantified. For comparison, the transcriptomics analysis of the same cells identified a very similar number of (78) DUB mRNAs (Pinto-Fernández et al., 2019).

One of the limitations of the ABPP assay is the relatively low throughput due to the complexity of the sample preparation in proteomic applications. Here, we developed methodology to apply activity-based protein profiling in conjunction with enzyme inhibitors in a high-throughput manner, allowing for rapid screening of the concentration dependence and selectivity of multiple inhibitors simultaneously. Although this workflow can be implemented for any ABP containing an affinity tag motif, we used ubiquitin based ABPs to screen cysteine protease deubiquitylating enzymes (DUBs) and a panel of small molecule inhibitors as a methodological proof of concept.

## Materials and Methods

### Cell Culture and Lysis

MCF-7 cells were cultured in Dulbecco’s Modified Eagle’s Medium (DMEM) with high glucose and supplemented with 10% (v/v) Fetal Bovine Serum. SH-SY5Y cells were cultured in Eagle’s Minimum Essential Medium and Ham’s F12 Nutrient Mix (1:1), supplemented with 15% (v/v) Fetal Bovine Serum, 1% (v/v) non-essential amino acids and 2 mM Glutamax. Cells were maintained at 37°C, 5% CO_2_.

For cell collection and lysis, cells were washed with phosphate-buffered saline (PBS), scraped in fresh PBS and collected at 300 × g. Cells were resuspended in lysis buffer (50 mM Tris Base, 5 mM MgCl_2_.6 H_2_O, 0.5 mM EDTA, 250 mM Sucrose, 1 mM Dithiothreitol (DTT), pH 7.5) and vortexed with an equal volume of acid washed glass beads 10 times for 30 s, with 2 min breaks on ice. MCF-7 cell lysates were clarified by 14,000 × g centrifugation at 4 °C for 25 min. SH-SY5Y cell lysates were clarified at 600 × G at 4 °C for 10 min to retain USP30 bound to mitochondria. Protein concentrations were determined by BCA protein assay. Chemicals and reagents used in this study are summarised in Table 1.

**TABLE 1:** List of reagents.

Reagent	Source	Identifier
Antibodies		
HA (12CA5)	Roche	11666606001
USP7 (mouse)	Sigma-aldrich	May-46
USP7 (human)	ENZO	BML-PW0540-0100
GAPDH	Invitrogen	MA5-15738
USP30	Abcam	ab235299
Chemicals, Kits, Enzymes, and Others		
Sucrose	Alfa aesar	A15583
Acid washed glass beads	Sigma-aldrich	G4649
Sodium chloride	Sigma-aldrich	S5886
*Glycine*	Sigma-aldrich	G7126
Tween 20	Sigma-aldrich	P1379
Dulbecco’s modified eagle medium - high glucose	Gibco	11995040
Eagles minimum essential medium	Sigma-aldrich	M2279
Ham’s F12 nutrient mixture	Sigma-aldrich	N4888
Non-essential amino acids	Sigma-aldrich	M7145
Glutamax	Gibco	35050061
Fetal bovine serum	Gibco	10500064
ethanol	Merck life science	32221
Tris base (trizma)	Sigma-aldrich	T1503
Magnesium chloride	Sigma-aldrich	M2670
EDTA	Fischer bioreagents	6381-92-6
TFA	Sigma-aldrich	74564-10ML-F
Urea	Sigma-aldrich	U1250
NP-40	Sigma-aldrich	I3021
TEAB	Sigma-aldrich	T7408
PBS	Sigma-aldrich	D8537
SDS	Sigma-aldrich	71725
phosphoric acid	Fluka	79620
formic acid	Sigma-aldrich	33015
Methanol	Merck life science	32213
Iodoacetamide	Sigma-aldrich	I1149
Dithiothreitol	Sigma-aldrich	D9779
Acetonitrile	Millipore	1000302500
dimethyl sulfoxide	Sigma-aldrich	34869
Water for chromatography	Millipore	1153332500
BCA protein assay kit	Thermo scientific	23227
Trypsin	Worthington	LS003740 TPCK-treated
PA-W cartridges	Agilent	G5496-60000
Evotips	Evosep	EV-2001
S-trap plate	Protifi LCC	C02-96well
96-Well microplate, U-bottom	Greiner bio-one ltd	650201
Experimental Models: Cell Lines		
MCF-7	ATCC	HTB-22
SH-SY5Y	ATCC	CRL2266
*INSTRUMENTS*		
Nanodrop	ND-1000 spectrophotometer	1,000 3.8.1
Software		
Maxquant	MPI, www.maxquant.org	Versions 1.6.10.43 and 1.6.14
Sample prep workbench	Agilent	Version 3.0.0
Vworks	Agilent	Version
Graph pad prism	Prism 8	Version 8.4.3 (686)
Excel	Microsoft office 365	

### DUB Small Molecule Inhibitors Used in This Study

USP7 inhibitors FT671 and FT827 (Ioannidis et al., 2016; Turnbull et al., 2017) were a kind gift from Stephanos Ioannidis. USP30 inhibitor 39 (Kluge et al., 2018), and USP30 inhibitor 3-b (patent WO2020072964) were kindly provided by Jeff Schkeryantz and Lixin Qiao (Evotec/Bristol-Meyers-Squibb). Inhibitor structures are shown in Supplementary Figure S3. PR619 was purchased from Calbiochem (Cat. No. 662141), N-Ethylmaleimide from Sigma-Aldrich (Cat. No. E3876), USP7 inhibitor P22077 from Calbiochem (Cat. No. 662142), and USP7 inhibitor HBX41108 from TOCRIS (Cat. No. 4285).

### Tissue Collection and Lysis

Tissue was harvested from mice culled by exsanguination under terminal anesthetic (isoflurane > 4% in 95% O_2_ 5% CO_2_); depth of anesthesia was monitored by respiration rate and withdrawal reflexes. Mice were perfused with PBS and tissue frozen at −80°C. Mouse brain was homogenized in lysis buffer used for the lysis of cultured cells, using a dounce homogenizer. Once the tissue reached a homogenous consistency the glass bead lysis protocol was carried out as outlined for cultured cells. Lysates were clarified at 600 × g at 4°C for 10 min.

### Western Blotting

Samples were boiled in Laemmli sample buffer and separated on a Tris-glycine SDS page (4–15% acrylamide gradient) gel. Samples were then transferred to a PVDF membrane and blocked for 1 h in 4% milk TBST. Primary antibodies were incubated overnight at 4°C and secondary antibodies were incubated for 1 h at room temperature. Imaging was carried out on a LI-COR odyssey detection system.

### Probe Synthesis

HA-Ub-PA was synthesized as previously described (Borodovsky et al., 2002; Pinto-Fernández et al., 2019). Ubiquitin was expressed in *E. coli* (Gly76del) with an N-terminal HA tag and a C-terminal intein-chitin binding domain (CBD). *E. coli* were suspended in 50 mM Hepes, 150 mM NaCl, 0.5 mM DTT (buffer used throughout synthesis) and sonicated 10 times, 30 s on, 30 s off. Purification was carried out using Chitin bead slurry, and HA-Ub-MesNa was formed via overnight agitated incubation with 100 mM MesNa at 37 °C while the protein was still attached to the Chitin beads. HA-Ub-PA was formed by incubation of HA-Ub-MesNa with 250 mM PA with agitation at room temperature for 20 min. Excess PA was removed via PD-10 column desalting. Complete and active probe formation was confirmed via anti-HA western blot and intact protein LC-MS (data not shown).

### Probe and Inhibitor Labeling

Lysates were diluted to 3.33 mg/ml using lysis buffer (minus the volume of the inhibitor and probe). Inhibitors were diluted with either DMSO (or ethanol in the case of NEM) to the same volume for their concentration dependence. 3-b, 39, FT671 and NEM were incubated with cell/tissue lysates for 1 h at 37°C. Probe was incubated with lysate at a ratio of 1:200 (w/w) for 45 min at 37 °C in all conditions, except for FT671 (10 min incubation) due to long probe incubations displacing bound inhibitor in this case. Reactions were quenched by addition of SDS to 0.4% (w/v) and NP40 to 0.5% (v/v) and made to 1 mg/ml protein concentration by addition of NP40 buffer (50 mM Tris, 0.5% NP40 (v/v), 150 mM NaCl, 20 mM MgCl_2_, pH 7.4) (freezing at this point had no effect on the result of the subsequent IP).

### Immunoprecipitation With Agilent Bravo AssayMAP Liquid Handling Robot

Anti-HA (12CA5) antibody (Roche) was immobilized on Protein A (PA-W) cartridges (Agilent, G5496-60000), using the Immobilization App (Agilent Sample Prep Workbench v3.0.0). All steps use PBS buffer (Sigma-Aldrich). Cartridges were primed with 100 μL (at 300 μL/min) and equilibrated with 50 μL (at 10 μL/min) followed by loading of 100 μg antibody (or otherwise as stated) in a final volume of 50 μL PBS buffer at 3 μL/min. A cup wash with 50 μL and an internal cartridge wash step (100 μL at 10 μL/min) were performed before re-equilibrating the cartridges with 50 μL at 10 μL/min).

The Affinity Purification App was used for pull-downs. Briefly, Protein A cartridges with immobilized anti-HA antibody were primed (100 μL at 300 μL/min) and equilibrated (50 μL at 10 uL/min) with NP-40 buffer, which was also used for all following steps. The sample was loaded at a flow-rate of 1 μL/min. After sample loading the cup was washed (50 μL) and an internal cartridge wash step (100 uL at 10 μL/min) performed to remove unbound lysate. Peptides were eluted using 50 μL at 5 μL/min 6M Urea or 0.15% TFA or 5% SDS.

### Mass Spectrometry Sample Preparation and Analysis

Urea and TFA eluates were diluted/neutralized with 180 μL 100 mM TEAB. Samples were digested in solution with 1 μg Trypsin (Worthington, LS003740 TPCK-treated Trypsin) over night at 37 C. Digestion was stopped by acidification to final concentration of 1% formic acid.

SDS eluates were prepared following an S-Trap 96-well plate (Protifi LLC, C02-96well) protocol. Eluates were acidified with ∼12% phosphoric acid (10:1 v/v) and loaded into the S-trap containing 350 μL 90% MeOH in 100 mM TEAB and spun at 1,500x g for 1 min. This step was repeated three times. Then samples were resuspended in 100 μL 100 mM TEAB with 1 μg Trypsin (Worthington) and digested over night at 37 C. Samples were eluted from the S-traps in three consecutive steps, each for 1 min at 1,500 × g, first with 50 μL 50 mM TEAB, then 50 μL 0.1% TFA and finally 50 μL 50% ACN/0.1% TFA. The combined eluates were dried down in a vacuum centrifuge and resuspended in 2% ACN/0.1% TFA for LC-MS.

### LC-MS/MS Data Acquisition

Samples were either run on a liquid chromatography tandem mass spectrometry (LC-MS/MS) setup comprised of an Evosep OneLC coupled to a Bruker timsTOF Pro mass spectrometer or a Dionex Ultimate3000LC coupled to a Thermo Q Exactive Classic orbitrap mass spectrometer.

Evotips (Evosep) were prepared and loaded with peptides as described by the manufacturer. Briefly, Evotips were activated by soaking in isopropanol and primed with 20 µL buffer B (ACN, 0.1% FA) by centrifugation for 1 min at 700 g. Tips were soaked in isopropanol a second time and equilibrated with 20 µL buffer A (water, 0.1% FA) by centrifugation. 20 µL buffer A were loaded onto the tips and the samples were added. Tips were spun and then washed with 20 µL buffer A followed by overlaying the C18 material in the tips with 100 µL buffer A and a short 20 s spin.

Peptides were separated on an 8 cm analytical C18 column (PepSep, EV-1109, 3 µm beads, 100 µm ID) using the pre-set 100 samples per day gradient on the Evosep One. MS data was acquired in PASEF mode (oTOF control 6.2.105/HyStar 5.1.8.1) in a mass range of 100–1700 m/z with four PASEF frames (3 cycles overlap). The ion mobility window was set from 1/k0 0.85 to 1.3 V/cm^2^, ramp time 100 m s with locked duty cycle.

On the Orbitrap setup comprised of a Dionex Ultimate 3,000 nano LC with Thermo Q Exactive Classic peptides were separated on a 50-cm EasySpray column (Thermo Fisher, ES803, 2 µm beads, 75-µm ID) with a 60 min gradient of 2 to 35% acetonitrile in 0.1% formic acid and 5% DMSO at a flow rate of 250 nL/min.

MS1 spectra were acquired with a resolution of 70,000 and AGC target of 3e6 ions for a maximum injection time of 100 m s. The Top15 most abundant peaks were fragmented after isolation with a mass window of 1.6 Th at a resolution of 17,500 with a maximum injection time of 128 m s. Normalized collision energy was 28% (HCD).

Data for experiment in Supplementary Figure S1D was generated as follow: Samples of the lysate titration on cartridge have been run on a timsTOF Pro (OtofControl 6.0.115/HyStar 5.0.37.1) coupled to a Dionex Ultimate 3,000 on a 15 cm IonOpticks Aurora series column (1.6 µm beads, 75 µm ID, Ionopticks AUR2-15075C18A) at a flow rate of 400 nL/min. The gradient started for 3 min at 2% B increasing linearly in 17 min to 30% B followed by ramping up to 95% for 1 min and re-equilibration to 2% B. Data has been acquired in PASEF mode as described above.

Data in Figure 2 with compounds FT671, FT827, HBX41108, P22077, 3-b, 39, and PR619 has been acquired on a 100 samples per day gradient on a 8 cm Pepsep column (1.5 µm beads, 150 µm ID, PepSep, PSC-8-150-15-UHP-nC) with PASEF data acquisition as described above.

### MaxQuant Analysis

Orbitrap raw data was searched in Maxquant 1.6.10.43, timsTOF data was searched in Maxquant 1.6.14. MCF-7 and SHSY5Y cell samples against a reviewed *Homo sapiens* Uniprot database (retrieved 31-Dec 2018), mouse brain against a reviewed *Mus musculus* Uniprot database (retrieved 17-Oct 2020).

Maxquant default settings have been used with oxidation of methionine residues and acetylation of the protein N-termini set as variable modifications and carbamidomethylation of cysteine residues as fixed modification. The match between runs feature was used for all analyses.

Raw data and Maxquant search results have been deposited to PRIDE with the identifier PXD023036.

### Data Analysis

Graphs were generated and fitted using Graphpad Prism version 8.4.3 (686). All intensities from mass spectrometry experiments are LFQ (label-free quantitative) intensities unless otherwise stated. DUBs were filtered and removed based off presence in a no probe control sample, missing values in the probe control sample, or intensity values that were at the bottom limit of the MS dynamic range.

## Results

### ABBP-HT Workflow Optimization

**FIGURE 1:**
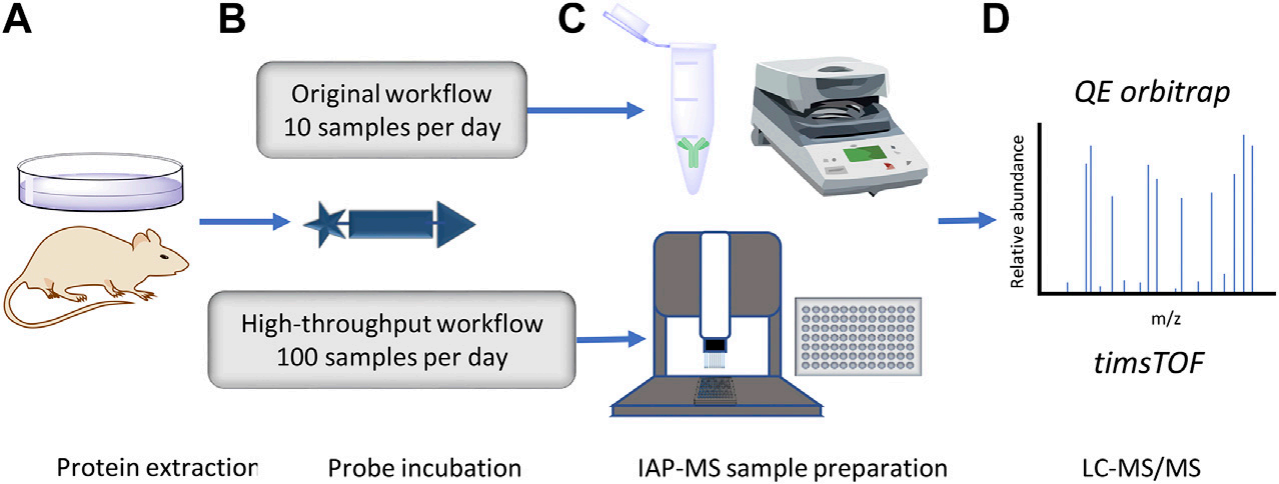
Accelerated DUB inhibitor ABP IP workflow. **(A)** Protein extraction and inhibitor treatment of either intact or lyzed tissue/cell lines. **(B)**. HA-Ub-PA probe incubation to label uninhibited cysteine active DUBs. **(C)**. Anti-HA IP, traditionally with centrifugation or magnetic collection of agarose beads in a low throughput format. In this work the throughput is increased to a 96 well plate format using an Agilent bravo liquid handling platform. **(D)**. LC-MS/MS proteomic analysis of immunoprecipitated DUBs. Here we compare the depth of the DUBome obtained using a QE orbitrap vs. a high-throughput timsTOF.

Each stage of the high-throughput IP methodology outlined in Figure 1 was optimized for maximal DUB identification coverage with minimal material to reduce experimental cost/time. Different starting material type and concentration, probe labeling, immunoaffinity purification, elution, and sample preparation conditions were tested and optimized.

First, we decided to identify the ideal ratio of probe to protein in the lysate by incubating increasing concentration of the probe with a fixed amount of lysate. Note: this step should be carried out every time after changing the batch of ABP. Our results (shown in Supplementary Figure S1D) suggested that we should use at least 0.25 µg of HA-Ub-PA probe to label efficiently 50 µg protein extracts from two different cell lines, MCF-7 and SH-SY5Y.

In parallel, we also determined the best antibody concentration for immunoprecipitation. Anti-HA antibody was loaded onto Protein A cartridges as a set volume of 50 μL at a flow rate of 3 μg/uL. The column was then washed with 100 μL of PBS buffer. From this, the loading and washing flow through fractions were collected, and unbound antibody was detected by 280 nm Nanodrop measurements. While residual amounts of protein were detected at low concentrations, a significant amount of protein was present when 90 μg was loaded, suggesting the column saturates between 80 and 90 μg of antibody (Supplementary Figure S1B). From this, we concluded that above 80–90 μg of antibody saturates all column binding sites.

Using cartridges primed with 80 μg of HA antibody, a concentration dependence was also carried out using varying amounts of HA-Ub-PA probe which was diluted to a set volume of 25 μL and loaded at a flowrate of 3 μL/min. The presence of probe in the loading and washing flow-through fractions was detected by anti-HA immunoblotting. Unbound probe was detected both in loading and washing flow-through fractions when ≥5 μg of probe was flowed through the column (Supplementary Figure S1C). Therefore, no more than 2–5 μg of probe should be loaded to avoid column saturation and material waste.

**FIGURE 2:**
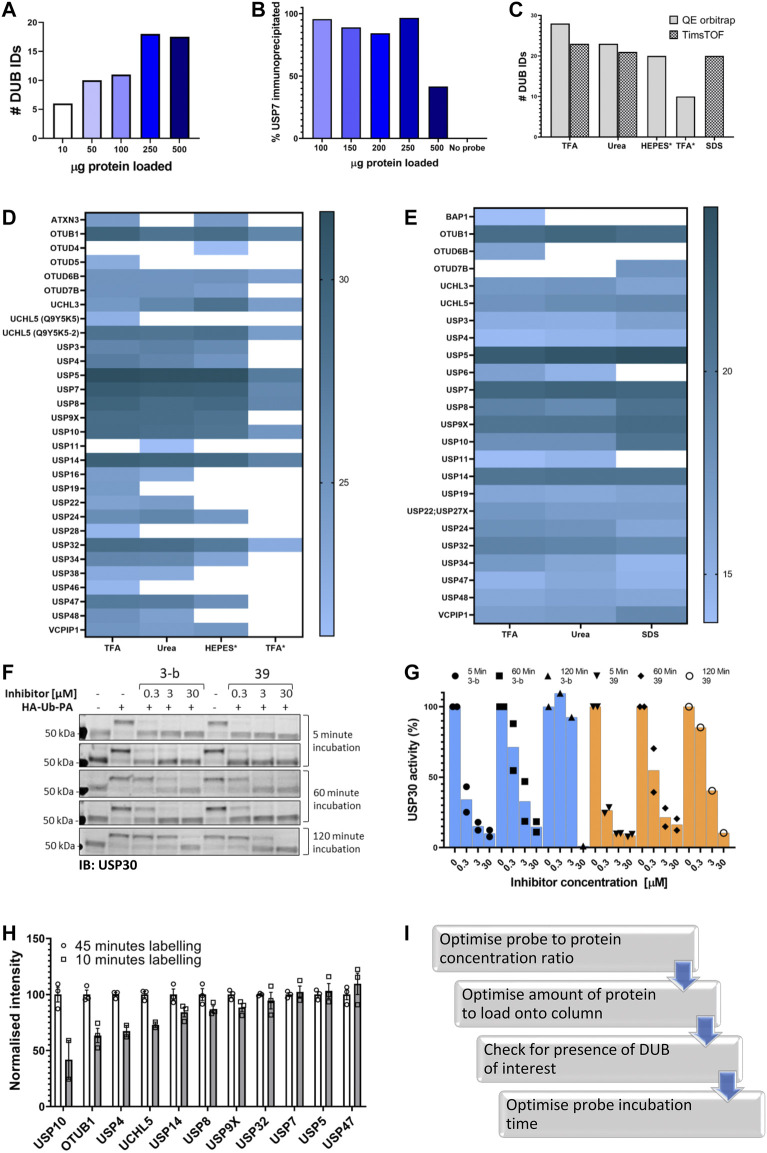
Optimizing the ABPP-HT workflow. **(A)**. Number of DUBS identified by timsTOF MS with increasing amounts of HA-Ub-PA-labelled MCF-7 lysate protein, after immunoprecipitation and elution with 0.15% TFA. **(B)**. Western blot densitometry quantification (full blot in Figure S2D) of USP7 in the immunoprecipitation loading flow-through, with increasing amounts of HA-Ub-PA-labelled MCF-7 lysate protein quantity immunoprecipitated and eluted with 0.15% TFA. **(C)**. Number of DUBs identified by LC-MS/MS with different IP elutions: 0.15% TFA, 6 M urea, HEPES, * = On column digestion. **(D)**. Log2 intensities of DUBS identified with different elution methods by a QE orbitrap MS. 0.15% TFA, 6 M urea, HEPES, * = On column digestion. **(E)**. Log2 intensities of DUBs identified with different elution methods by a timsTOF MS. 0.15% TFA, 6 M urea, 5% SDS. **F**. USP30 immunoblots showing mouse brain lysate displacement of a covalent (3-b) and non-covalent (39) USP30 inhibitor with increasing HA-Ub-PA (at 37°C) incubation times. **(G)**. The densitometric quantification of Figure 3F from the intensity of the HA-Ub-PA-labelled band, normalized to the intensity of both USP30 bands together. **(H)**. timsTOF DUB intensities of MCF-7 labeled with HA-Ub-PA for 10 min normalized to 45 min at 37°C (SEM, *n* = 3). **I**. Optimization workflow for high-throughput DUB inhibitor screening using ABPP LC-MS/MS.

**FIGURE 3:**
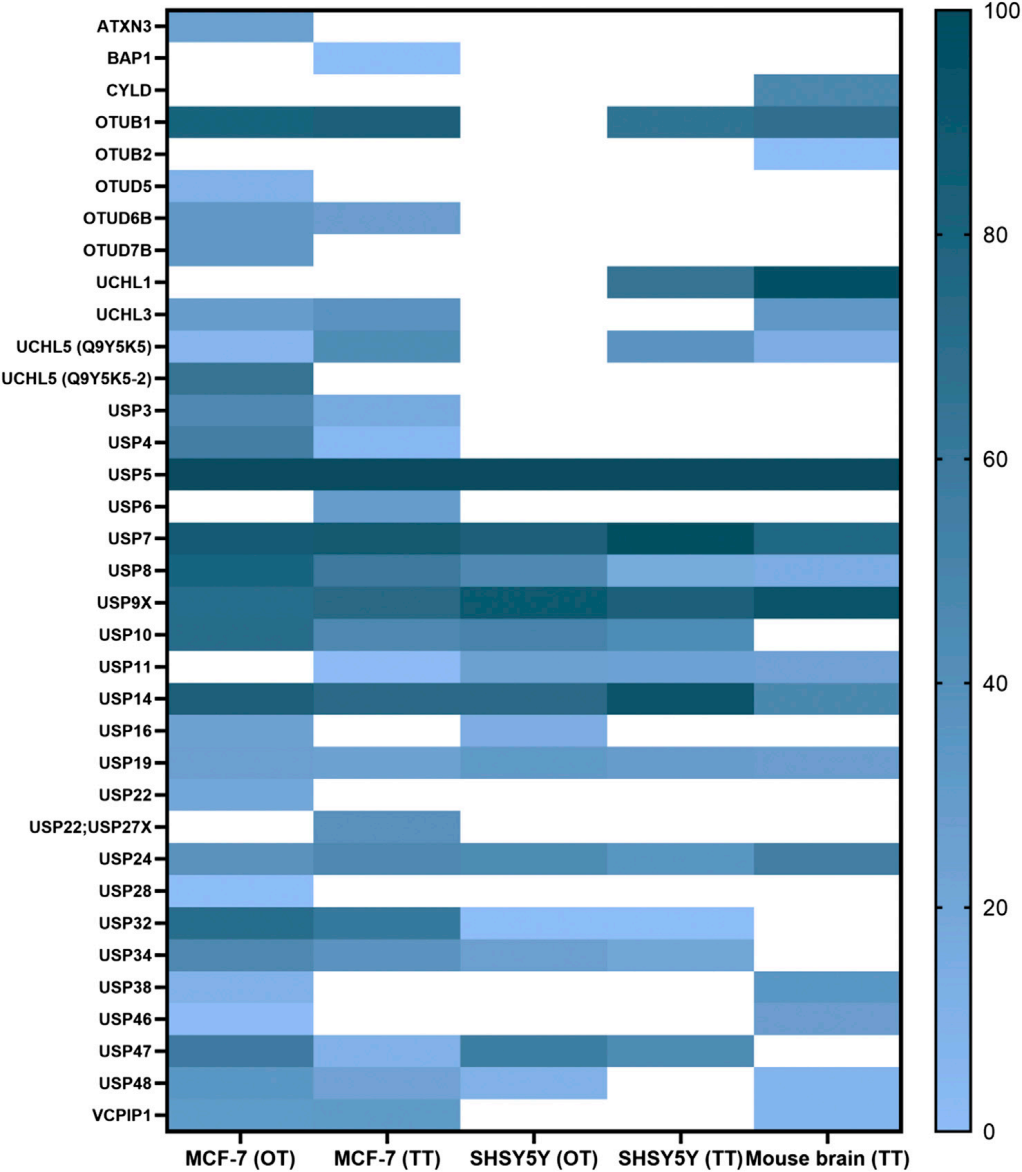
ABPP-HT reveals cell type-specific DUB profiles. DUB intensities as determined by HA-Ub-PA activity-based probe profiling (ABPP) and identified from different cell types and tissue quantified by mass spectrometry using either a QE orbitrap (OT) or timsTOF (TT), normalized within each dataset.

In order to identify the optimal amount of labeled lysate we performed an LC-MS/MS analysis after performing IAP-MS with different amounts of labeled lysate, using the parameters described above. The results (Figure 2A) showed that 250 µg of labeled lysate gives us the maximum of DUB identifications (IDs). Analysis of the flow through by immunoblotting of USP7 confirmed these results (Figure 3B, Supplementary Figures S1D and S2).

The next step was to optimize the elution of the immunoprecipitated material from the column, the digestion method, and the LC-MS/MS instrumentation. Various elution/digestion methods were trialed. Initially 6 M Urea or 0.15% TFA were used to elute the proteins for digestion and MS analysis. An on-column trypsin digestion of the protein was also carried out in the presence of either HEPES buffer or 0.15% TFA. Samples were then run on a Q Exactive Orbitrap mass spectrometer and quantified using the search software Maxquant. From this, the most efficient elution was 0.15% TFA in combination with in-solution trypsin digestion. Different elution methods followed by in-solution digestions were then trialed on an Evosep (liquid chromatography; LC) and timsTOF Pro (mass spectrometry; MS). Comparison of 5% SDS, 0.15% TFA and 6M urea using this instrumentation demonstrated that 0.15% TFA is again the most efficient elution for the identification and quantification of the highest number of DUBs (Figures 2C–E).

The combined use of short gradients on an Evosep LC and the fast scan speeds using Parallel Accumulation Serial Fragmentation (PASEF) data acquisition on a timsTOF Pro allowed for increased sample throughput. With the preset gradients on the Evosep throughput ranges from 30 to 300 samples per day (SPD), compared to the 6-12 SPD of common nanoflow LC setups or nine SPD on our in-house 1 h gradient. In this experimentation 100 SPD is used as standard, with no increase in DUBs identified occurring from a 60 SPD method (data not shown). Additionally, comparison of the Evosep One with a nanoflow LC coupled to the timsTOF Pro resulted in no marked difference in the number of identified DUBs (23 *vs* 22 respectively) on comparable gradient lengths. There is ∼20% reduction in the number of DUBs identified with the TFA elution using the Evosep/timsTOF compared to an Orbitrap MS (Figures 2D,E) at highly reduced instrument time (single run ∼15 min vs 160 min). Both instruments lead to the identification of a similar panel of DUBs, with some DUBs unique to each. The choice between the two instruments should balance the required DUBome coverage *vs* throughput, and whether detection of the desired DUB is feasible with the chosen methodology.

Finally, since one of the applications of this methodology is DUB inhibitor characterization there is an aspect of the ABP assay that should considered before testing a given inhibitor: Probe incubation time. Ub-based activity-based probes, especially with the highly reactive PA (propargylamide) warhead can displace both, covalent and non-covalent, DUB inhibitors over time. As shown in Figures 2F and 2G for two different USP30 inhibitors, the reversible covalent 3-b and the reversible non-covalent 39. Increasing the incubation time displaced the inhibitors, especially 3-b, from the DUB, giving the impression that the inhibitor is less potent. Therefore, for reversible inhibitors we suggest minimizing incubation times with the probe. Of course, this has an impact on the number of DUBs identified when performing the ABPP assay. We compared two labeling times using our ABPP-HT workflow and as expected, the intensities of some DUBs are clearly reduced when the lysate is incubated with probe for a shorter period of time (10 min vs. 45; Figure 2H). These optimization steps were summarized in Figure 2I.

### Guide to DUB Picking: Abundance Changes With Methodology and Starting Material Source

These conditions were then applied to characterize the active DUBome in two different cell lines, MCF-7 and SH-SY5Y, and brain tissue material from mice. We also included the data using the two different LC-MS/MS instrumentations: Nanoflow liquid chromatography coupled to an Orbitrap MS (OT on the figures) and microflow (Evosep) liquid chromatography and ion mobility-mass spectrometer, timsTOF (TT on the figures). We summarized the results in a heat map (Figure 3), displaying the normalized intensities of the identified DUBs when using different cell lines, tissue, and instrumentation. This together with the heat maps describing the different elution methods (Figures 2D,E) should be a good reference when studying a particular DUB and its potential inhibitors. For example, there are some DUBs that are only identified in the cancer cells like USP3, USP4, and OTUD7B. On the other hand, there are DUBs specific for brain tissue and cells like UCHL1.

### Proof of Concept: Broad and Specific DUB Inhibitor Concentration Dependences and Cross-Reactivities

**FIGURE 4:**
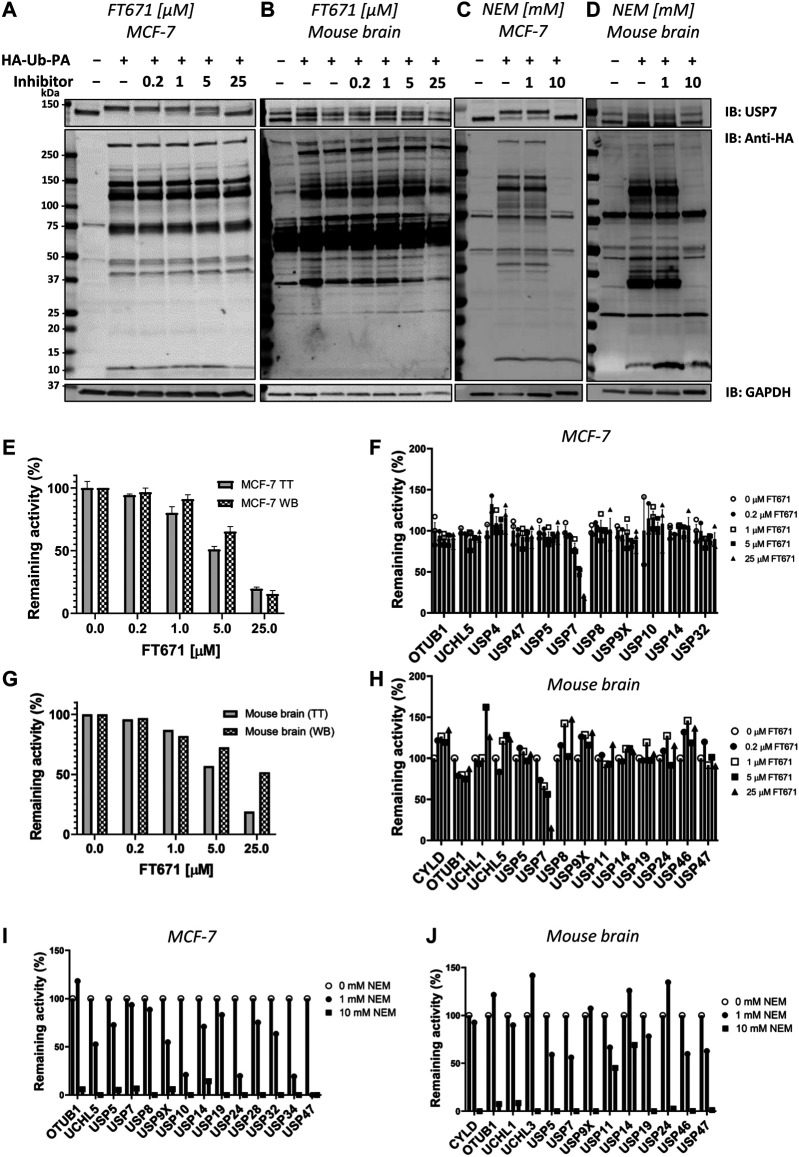
ABPP-HT allows fast generation of DUB inhibitor selectivity profiles in a cellular context. **(A)–(D)**. USP7, HA, and GAPDH (loading control) immunoblots of a concentration dependence of FT671 USP7 specific inhibitor and NEM (a general cysteine modifier) in mouse brain tissue and MCF-7 cell lysates. **(E)**. The densitometric quantification (WB) of three independent experiments as in Figure 5A from the intensity of the HA-Ub-PA USP7 MCF-7 labeled band (SEM, *n* = 3), normalized to the intensity of both USP7 bands together, compared to the MCF-7 timsTOF LFQ normalized intensity (TT) of immunoprecipitated USP7 (MS data SEM *n* = 3 (for 0.2 μM, *n* = 2)). **(F)**. The activity of a panel of DUBS identified by timsTOF MS from MCF-7 with increasing concentration of FT671 (SEM *n* = 3 (for 0.2 μM, *n* = 2)). **(G)**. The densitometric quantification of Figure 5B from the intensity of the HA-Ub-PA USP7 mouse brain labeled band, normalized to the intensity of both USP7 bands together (WB), compared to the mouse brain timsTOF LFQ normalized intensity (TT) of immunoprecipitated USP7. **(H)**. The activity of a panel of DUBS identified by timsTOF MS from mouse brain with increasing concentration of FT671. **(I), (J)**. The activity of a panel of DUBS identified by timsTOF MS from MCF-7 and mouse brain lysates respectively, treated with either 1 or 10 mM of NEM.

The ABPP-HT methodology is able to identify a reduced but representative panel of DUBs when compared to the regular ABPP (∼15–25 vs. ∼30–40) (Pinto-Fernández et al., 2019). With this representative panel we applied the methodology to check for compatibility with DUB inhibitor characterization. In order to do so, we performed ABPP-HT with the highly selective USP7 inhibitor FT671 (Turnbull et al., 2017) and with the broad cysteine modifier NEM (n-ethylmaleimide) (Pinto-Fernández et al., 2019). We performed these experiments treating lysates from MCF-7 cells and from mouse brain tissue extracts with different concentrations of the inhibitors. We used the TT LC-MS/MS instrumentation as it is more suitable for testing a higher number of compounds. First, we performed control immunoblots against USP7, to show the effects of the compounds on USP7, and against the probe (anti-HA), to visualize the selectivity of the compounds. FT671 inhibits USP7 in both, cell line (Figure 4A) and brain tissue (Figure 4B), however, due to the USP7 antibody recognizing USP7 with and without its previously characterized ubiquitination (Fernández-Montalván et al., 2007), in the mouse tissue this effect was more challenging to visualize. At the same time, the HA blot showed little reactivity of FT671 with other labeled DUBs. On the other hand, NEM was also inhibiting USP7 in both cells (Figure 4C) and brain (Figure 4D), however, in a non-selective way as shown by the overall decrease in HA signal at high concentrations of the compound. These observations were highly comparable when processing these samples on our ABPP-HT workflow. For instance, immunoblot densitometry of labeled USP7 with increasing amounts of FT671 correlates to a similar degree with the LC-MS/MS data (MCF-7 on Figure 4E and brain on Figure 4G). This was also the case for the selectivity profile of the two compounds in both cells and brain (Figures 4F,H–J), reflecting well both, the expected high selectivity of FT671 and the broad inhibition by NEM.

### Exploiting the Possibilities of the ABBP-HT Methodology: Multiple Compound Characterization in Different Cell Lines and Tissue

**FIGURE 5:**
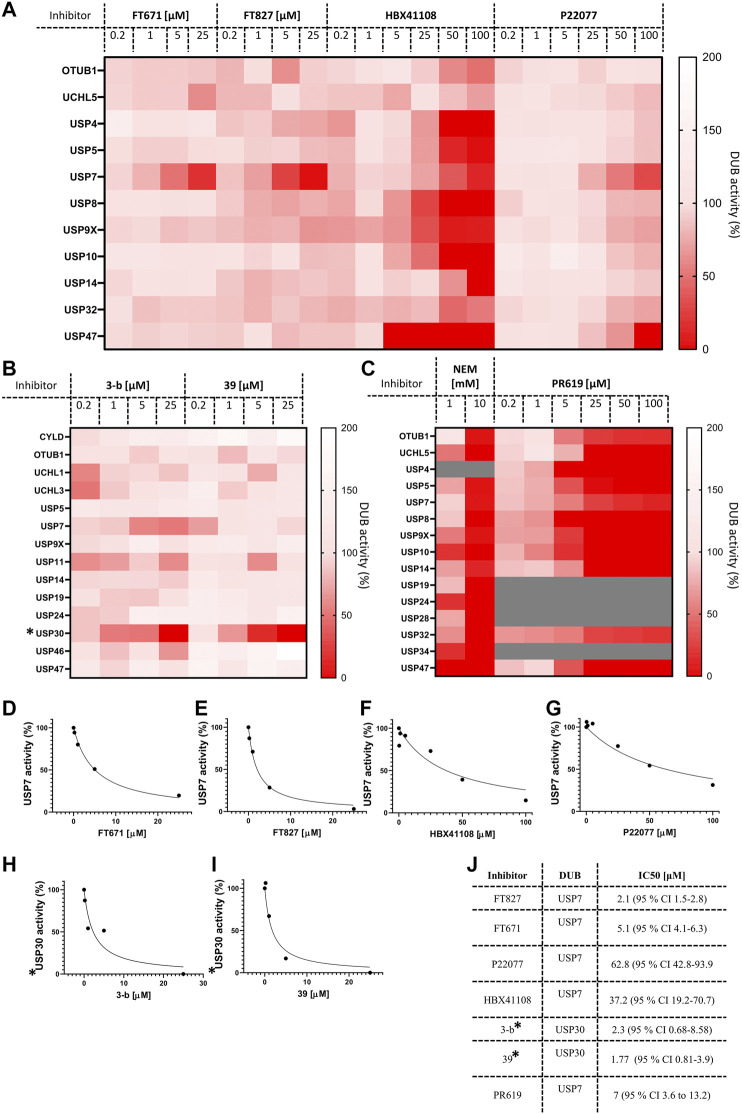
ABPP‐HT reveals DUB inhibitor selectivity and specificity compatible with higher throughput. **(A)**. The activity of a panel of DUBs from MCF‐7 identified from timsTOF MS, in response to USP7 specific inhibitors FT671 (n 3 (for 0.2 μM n 2)), FT827, HBX108 and P22077. **(B)**. The activity of a panel of DUBs from mouse brain lysate identified from timsTOF MS, in response to USP30 specific inhibitors 3‐b and 39. **(C)**. The activity of a panel of DUBs in MCF‐7 lysates identified by timsTOF LC‐MS/MS, in response to the cysteine modifier NEM, and broad spectrum DUB inhibitor PR619 (PR619 n 2). D‐I. From left to right concentration dependences of USP7 from inhibitors FT671, FT827, HBX41108, and P22077 in MCF‐7 lysates, and USP30 inhibitors for 3‐b and 39 in mouse brain. **(J)**. IC50 values extracted from D‐I, fit to equation: Y 100/(1 + X/IC50). * normalized raw intensities, not LFQ intensities.

Finally, we decided to gain advantage of the ABPP-HT possibilities and applied the methodology to a number of compounds (structures in Supplementary Figure S3) and concentrations simultaneously. Here, critical target engagement information was obtained in a cellular context, in a much faster way than the current methodology. We tested different concentrations of four USP7 inhibitors, FT671, FT827 (Turnbull et al., 2017), HBX41108 (Colland et al., 2009), P22077 (Altun et al., 2011), two USP30 inhibitors (3-b and 39), and the two broad cysteine modifiers NEM (Pinto-Fernández et al., 2019) and PR619 (Altun et al., 2011). The results are summarized on separated heat maps for USP7 inhibitors (Figure 5A), USP30 (Figure 5B) and non-selective (Figure 5C), and bar graphs (Supplementary Figures S4A–D). These results not only match the matching control immunoblots in Supplementary Figures S4E–H but also previously reported information. For instance, P22077 was reported to be a dual USP7/USP47 inhibitor (Altun et al., 2011) and the same result could be seen in our data (Figure 5A). FT671 and FT827 were reported to be highly selective, and potent, USP7 inhibitors (Turnbull et al., 2017) and this still applied when using our ABPP-HT workflow (Figure 5A). HBX41108 selectivity data has not been reported, although our results suggested a not very selective profile. The USP30 inhibitors showed a nice dose-dependent inhibition of the target and good selectivity profiles, especially for the USP30 inhibitor 39 (Figure 5B). Finally, the two cysteine modifiers behaved as expected (Altun et al., 2011) (Pinto-Fernández et al., 2019), inhibiting all the identified DUBs at high concentrations (Figure 5C). The ability to analyze multiple concentrations also allowed for the plotting and determination of the half-maximal inhibitory concentrations (IC50s) for each inhibitor in the described conditions (Figures 5D–J). Although not in a clear dose-dependent manner, the activity of some DUBs appears to increase in Figures 5A–C, this is likely attributable to a limiting probe concentration, which is increased as a consequence of DUB inhibition, leading to a complex alteration of the probes binding kinetics for other DUBs. It should be noted that in this methodology the binding capacity of the columns can be limiting, and while it is important to saturate the probe to lysate ratio as much as possible, it is also important to use as much lysate as possible to ensure a representative panel of DUBs are identified. Another possibility giving a similar effect would be when profiling a DUB inhibitor that binds to another off-target DUB but not at the catalytic site, inducing a conformational change in the enzyme that allows for better reactivity with the probe. Despite this being an interesting observation in the presence of some compounds, we believe that when interpreting the DUB profiling data, as shown in the heat maps in Figure 5, it remains important to infer cross-reactivity where intensity is affected in a clear inhibitor concentration-dependent manner.

## Discussion

There are numerous methods to study the potency and selectivity of an enzyme inhibitor using recombinant purified proteins, and their substrates, in biochemical assays. However, these assays cannot assess the activity of an inhibitor in a more relevant context such as cell lysates, intact cells or tissue. Degradation, limited permeability, or cross-reactivity of an inhibitor in the cellular environment may lead to reduced potency and off-target effects. Consequently, it is important to be able to screen potential inhibitors within this environment. ABPP assays can provide all of these very relevant parameters, however, if we want to apply this technique to a screen of inhibitors with varying concentrations in different cell types, the throughput needs to be increased.

Here, we describe a new ABPP methodology, named ABPP-HT (high-throughput-compatible activity-based protein profiling), that allowed the semi-automated analysis of samples in a microplate format, addressing the low throughput associated to the classic ABPP assay. The incorporation of a liquid handling robot compatible with IAP-MS, and the Evosep/timsTOF LC-MS/MS instrumentation, were key to boost the throughput of the ABPP up to ten times in a cost-effective way. While an Agilent Bravo liquid handling platform and an Evosep/timsTOF LC-MS/MS were applied here, the methodology could feasibly be applied to other liquid handling robots and fast scanning mass spectrometers.

**FIGURE 6:**
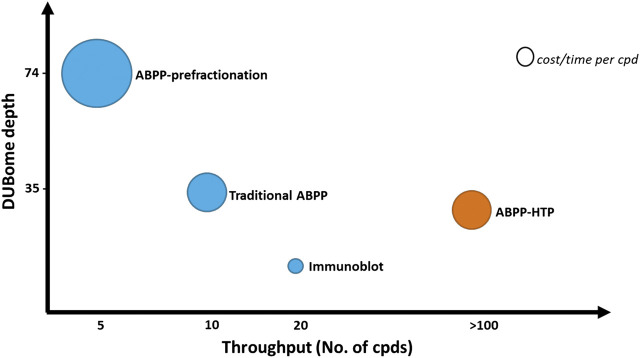
ABPP workflows optimized for DUBome depth and throughput. Comparison of cost and time between ABPP assays optimized for maximal coverage of cellular DUBs (DUBome depth–*Y*-axis) and higher-throughput approaches including traditional ABPP, immunoblot based ABPP and accelerated throughput ABPP (ABPP- HT) (Throughput–*X*-axis). Size of the circles are plotted represent relative cost per inhibitor compound tested. The cost and time per compound here are an estimate and will likely be reduced by the ABPP-HT workflow. However, this would be subject to individual instrument, reagent, and personnel accessibility/expenses.

The depth of this method is reduced when compared to the normal ABPP, with the detection of ∼15–25 DUBs acting as a representative panel when using the ABPP-HT vs. ∼30–40 DUBs with the original ABPP. The number of DUBs that are reactive with the probe and can be potentially detected by ABPP is higher than 70, but this requires performing a high-pH pre-fractionation of the samples prior LC-MS/MS analysis (Pinto-Fernández et al., 2019). This drastically increases the number of samples to analyze per condition and therefore the required time and cost of the assay. This comparative information has been summarized in Figure 6. The methodology of the ABPP-HT approach can be applied as a powerful initial screening tool for multiple inhibitors at different concentrations, in various cell lines, to discard weak or highly cross-reactive inhibitors quickly and robustly. From this, only potent and selective inhibitors could be taken forward for more thorough characterization using the original or fractionated ABPP approaches.

As a proof of concept, we demonstrated the versatility of this methodology using general and specific DUB inhibitors in two different cell lines and mouse brain tissue. ABPP-HT permitted the simultaneous analysis of six selective DUB inhibitors and the calculation of their respective half maximal inhibitory concentration (IC50) values. The methodology has the capacity to simultaneously test a much higher number of inhibitors and concentrations. We also believe that the throughput of the ABPP-HT can be increased even further. For example, by implementing chemical labels, such as TMT (tandem mass tag) that would allow the combination of up to 16 samples into one and therefore providing multiplexing capabilities and enhanced throughput. Another area where the sensitivity and therefore DUB coverage of this type of analysis could be further improved is by implementing targeted proteomics methods such as Data-Independent Acquisition (DIA) mass spectrometry. For instance, DIA has been successfully applied for ubiquitomics and discussed by Vere et al. (Vere et al., 2020).

In conclusion, ABPP-HT (high-throughput-compatible activity-based protein profiling) was conceptualized, optimized, and validated. When tested, the approach allowed for reduced time and cost for both sample preparation and MS time, while still identifying and quantifying a representative panel of endogenously expressed DUBs, enabling the profiling of a number of DUB inhibitors.

## Data Availability

The mass spectrometry proteomics data have been deposited to the ProteomeXchange Consortium via the PRIDE (Perez-Riverol et al., 2019) partner repository with the data set identifier PXD023036.
