# Supplementary material for: ABPP-HT - High-Throughput Activity-Based Profiling of Deubiquitylating Enzyme Inhibitors in a Cellular Context
Source: Front Chem. 2021 Feb 25;9:640105. doi: 10.3389/fchem.2021.640105 (PMC7947856; doi:10.3389/fchem.2021.640105)
Supplement: Supplementary file 1 [file image1.pdf]

Figure S1

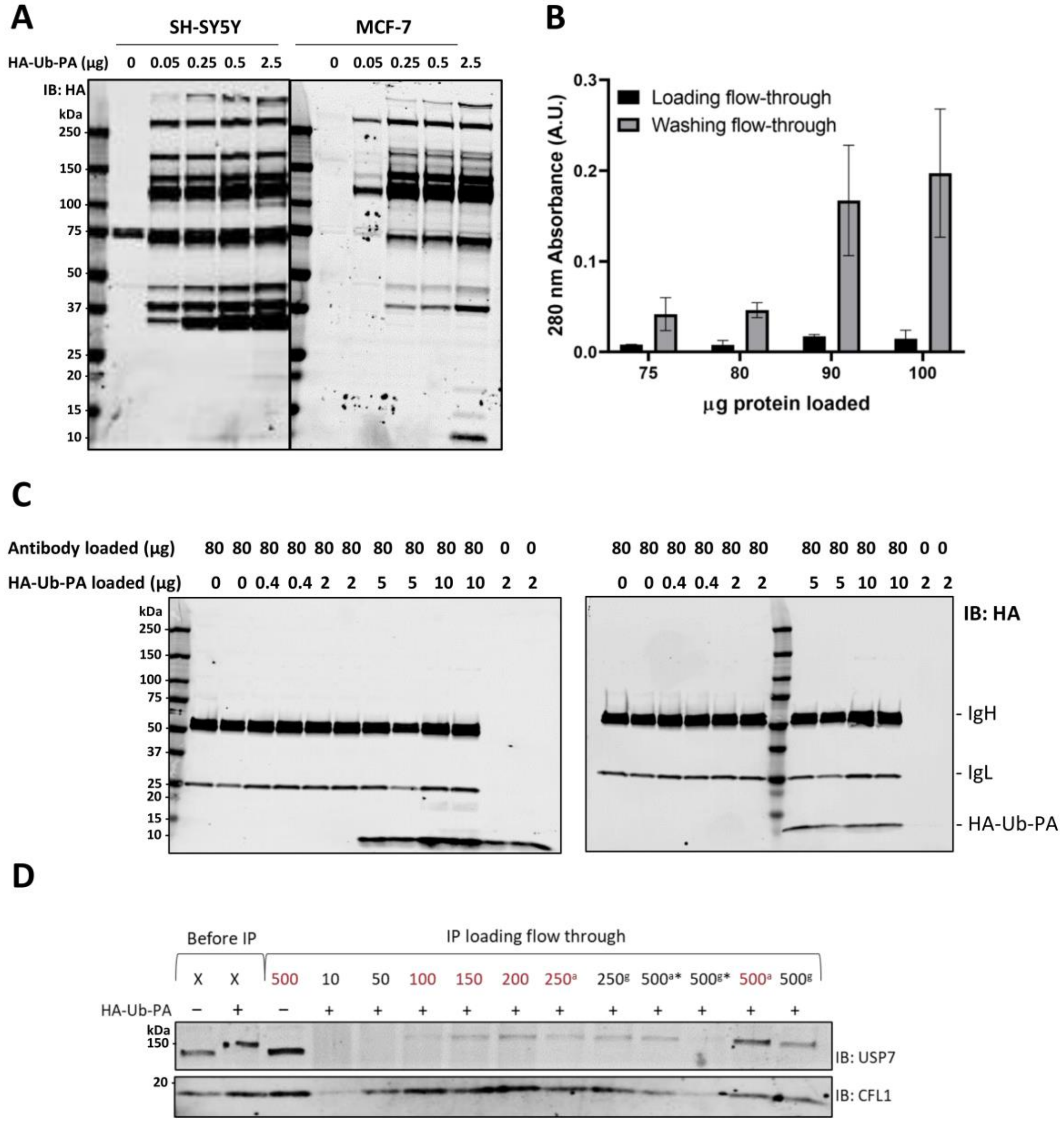

Figure S1. ***Optimizing DUB target engagement in the ABPP-HT workflow*** **A.** HA immunoblot of probe to lysate ratio optimisation, with 50 µg of either SHSY5Y lysate or MCF-7 lysate incubated with increasing amounts of HA-Ub-PA at 37 ˚C for 45 minutes. **B.** Concentration dependence of unbound antibody detected with antibody loading onto protein A columns. **C.** Concentration dependence of unbound HA-Ub-PA probe detected with HA-542 Ub-PA loading onto protein G column with 80 µg of antibody loaded. **D.** USP7 detected in IP flow-through where 100 µg of HA antibody has been loaded. Values in red were used for quantitation in figure 2B. a protein A column, g protein G column, *material was concentrated before loading to try to minimize loading time, the reduction in material is attributable to protein lost during the concentration step, not to increased HA-Ub-PA anti-HA binding. In each lane 5 ug of protein was loaded except in the case of 10 µg and 50 µg where samples were too dilute to load 5 µg.

Figure S2

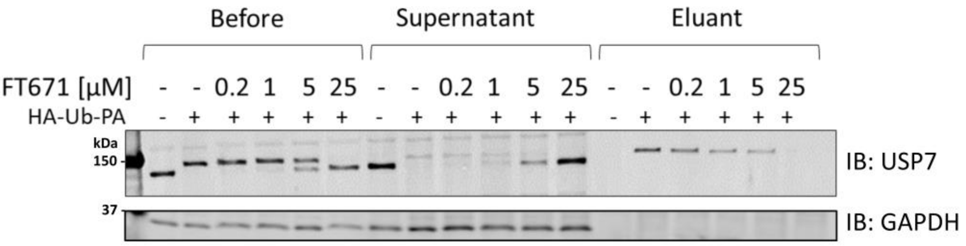

Figure S2. ***Efficient immunoprecipitation of USP7 with ABPP-HT*** USP7 immunoblot of FT671 concentration dependence. Before immunoprecipitation demonstrates probe labelling and inhibition, the supernatant and eluant bands show unlabelled and labelled bands respectively.

Figure S3

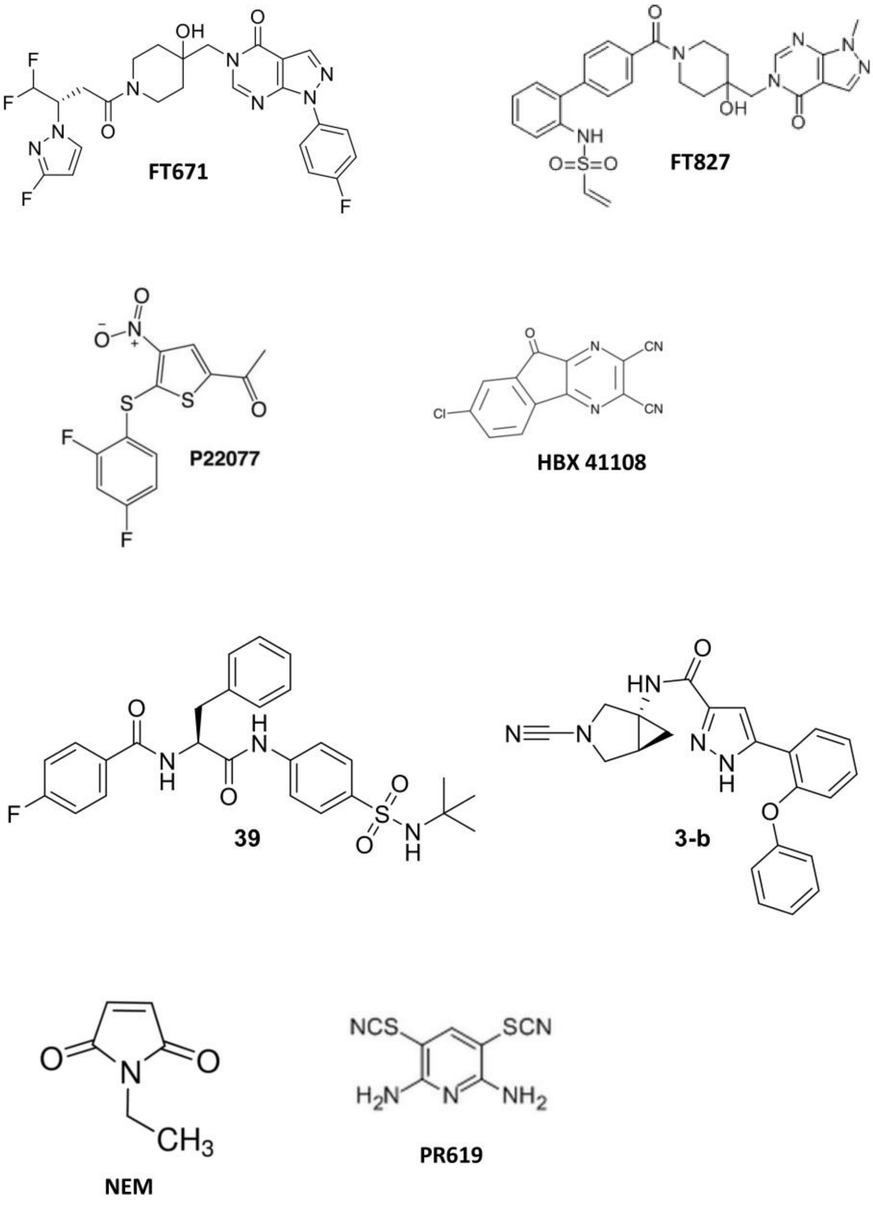

Figure S3. ***DUB inhibitors used in this study*.** USP7 inhibitors FT671 FT827, P22077, HBX 41108, USP30 inhibitor 39, USP30 inhibitor 3-b, and broad-spectrum cysteine modifiers NEM and PR619.

Figure S4

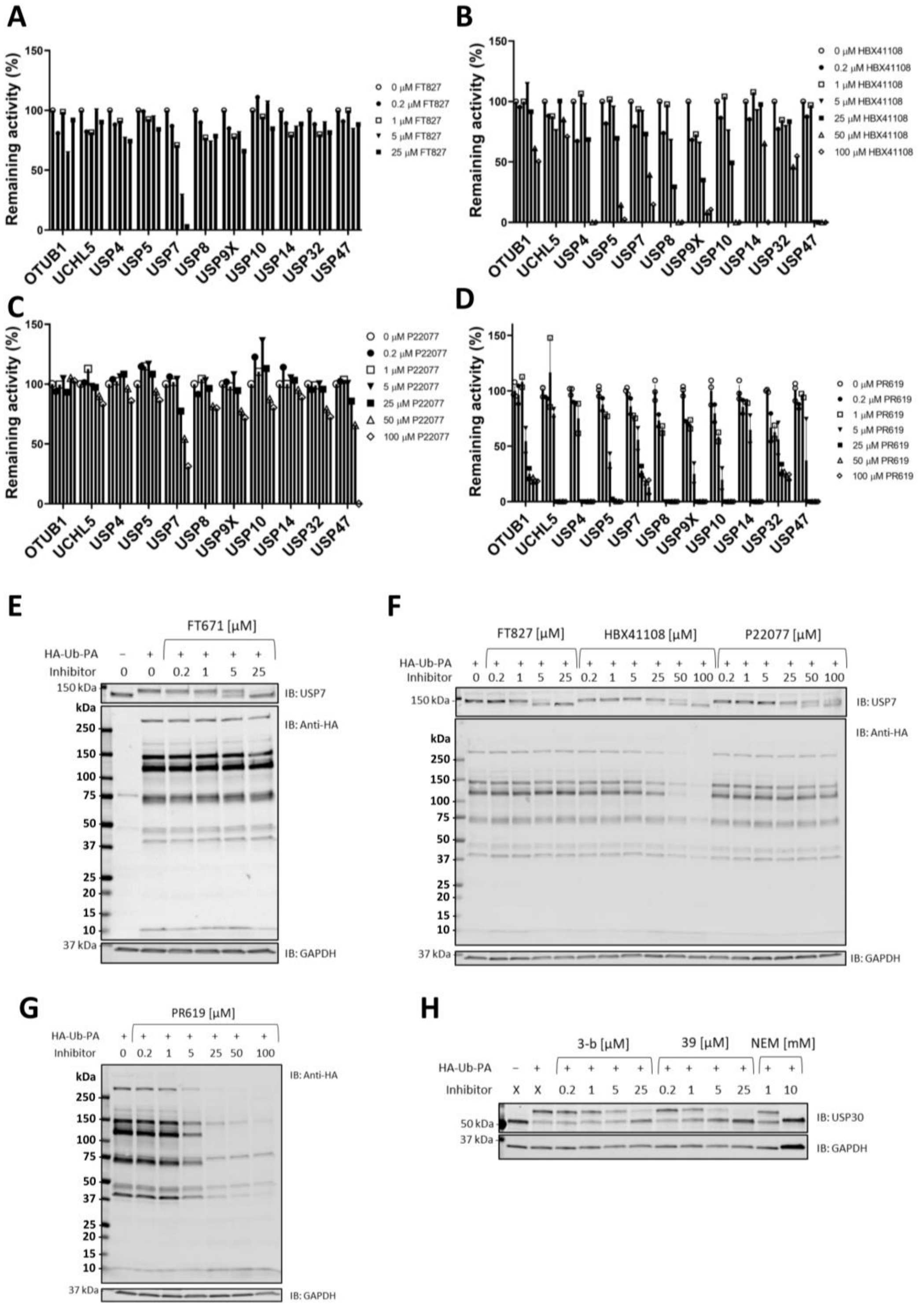

Figure S4. ***Inhibitor cellular target engagement assessed by ABPP*. A-D.** ABPP inhibition profile of a panel of DUBs by FT827, HBX41108, P22077, and PR609 (one experiment, except for PR609: SEM; n=2). **E.** USP7 and anti-HA immunoblot of specific USP7 inhibitor FT671. **F.** USP7 and anti-HA immunoblot of USP7 inhibitors FT827, HBX108 and P22077. **G.** Anti-HA immunoblot with broad-spectrum DUB inhibitor PR619. **H.** USP30 immunoblot of specific USP30 inhibitors 3-b and 39, and broad DUB inhibitor NEM in mouse brain lysates. GAPDH was used as loading control in these experiments.
